# An overview of vital functions of human ecdysoneless (ECD), a highly conserved protein

**DOI:** 10.1042/BSR20241270

**Published:** 2025-11-27

**Authors:** Kexin Liu, Xiankun Cao, Peixiang Ma, An Qin, Jie Zhao

**Affiliations:** 1 Shanghai Key Laboratory of Orthopedic Implants, Department of Orthopaedics Surgery, Shanghai Ninth People’s Hospital, Shanghai Jiao Tong University School of Medicine, Shanghai, 200011, China

**Keywords:** cancer, cell cycle, glucose metabolism, pre-mRNA splicing, transactivational regulation

## Abstract

Human ecdysoneless (ECD), the human ortholog of Ecd protein of *Drosophila melanogaster*, is a highly conserved protein. This protein comprises a well-folded N-terminal domain and a disordered C-terminal domain; these domains interact with multiple proteins and are involved in diverse biological processes, including cell cycle regulation, transactivation, pre-mRNA splicing, and glucose metabolism. ECD is highly expressed in various cancers, and its elevated expression is associated with poor prognosis in several types of human cancers. Therefore, it can serve as a potential biomarker and therapeutic target for treating tumors. This review focuses on the currently available knowledge of the physiological and pathological functions of ECD; moreover, the directions of prospective research in the relevant field have been discussed.

## Introduction

The protein ecdysoneless (ECD) was initially identified through the *ecd^1^* mutation in *Drosophila melanogaster*, which impaired steroid hormone (ecdysone) biosynthesis in larvae [1–4]. However, Redfern and Bownes [5] cautioned that numerous anomalies (such as the pupation process [1] and the protein synthesis in adipose tissue [6]) in *ecd^1^* stem from cell-autonomous requirements of Ecd for viability, implying these defects may not be solely attributable to ecdysone deficiency. In order to better explore the role of Ecd, Ivana Gaziova et al. identified the *ecd* gene [7]. The deduced amino acid sequence of Ecd demonstrated remarkable evolutionary conservation across species, which had been found to have 31% overall amino acid identity in humans [7]. They also observed that *ecd* ^−^ clones fail to survive in developing imaginal discs. Notably, Ecd was expressed in the ovary and functionally required in both somatic follicle cells and germline cells to support oocyte development [7]. These loss-of-function defects represented the first experimental demonstration of the cell-autonomous requirement for this evolutionarily conserved protein. During the period, the human ECD ortholog was identified for the first time in mammals through the screening of a human brain cDNA library and named human suppressor of GCR two (*SGT1*), which could rescue the deficiency of yeast mutants in glycolytic enzyme gene expression [8]. ECD exhibited broad tissue distribution in humans, yet its physiological significance remained elusive [8]. Sequence analysis identified several highly conserved motifs, none of which match established domain architectures in current databases [7,8]. Subsequently, the functions of ECD had attracted considerable attention in the past decades. Here, we summarize various properties and biological roles of ECD. (i) ECD interacts with the tumor suppressor p53 and stabilizes its protein levels. Moreover, it can interact with the ubiquitin ligase murine double minute-2 (Mdm2) to suppress the Mdm2-induced ubiquitination of p53 [9,10] or with thioredoxin-interacting protein (TXNIP) to compete with Mdm2, leading to the stabilization and increased activity of p53 [11]. (ii) Conditional deletion of *Ecd* in mouse embryonic fibroblasts induces a delay in the G1-S progression [12]. Furthermore, ECD participates in the regulation of the cell cycle also by interacting with the RuvB-like (RUVBL)1 component of the R2TP [RUVBL1-RUVBL2-RNA polymerase II-associated protein 3 (RPAP3)-PIH1 domain-containing protein 1(PIH1D1)] complex [13]. (iii) ECD plays a significant role in the transactivation. Despite the absence of a DNA-binding domain, the C-terminal region of ECD has transactivation activity, and it enhances transactivation activity by interacting with histone acetyltransferase p300 [14]. Our research found that Txnip interacts with the Ecd-p300 complex to regulate *Tnfsf11* transcription, thereby affecting aging and diabetes-induced osteoporosis [15]. ECD can bind to the components of mRNA export complexes and interact with the mRNA export-related DEAD BOX RNA helicase DDX39A to modulate the mRNA shuttle [16]. Moreover, Ecd plays a critical role in delivering spliceosome factor pre-mRNA processing 8 (PRP8; ortholog of mammalian PRPF8) to emerging U5 small nuclear ribonucleoprotein particles (snRNPs), which ensures splicing fidelity and transcriptome integrity [17–19]. (iv) During stress, ECD up-regulates the glucose-regulated protein 78 (GRP78) and enhances adaptive protein folding in the endoplasmic reticulum (ER) and further weakens downstream protein kinase RNA-like ER kinase (PERK) signaling, thereby promoting cell survival [20]. (v) Moreover, ECD is highly expressed and associated with signaling and metabolic pathways in many cancers [21–28]. Therefore, in this review, we briefly discuss available knowledge of the physiological and pathological functions of ECD. It is hoped that this will provide a more comprehensive introduction to the research progress of ECD and better explore the mechanisms related to cell growth, stress, metabolic aspects, and so on.

## ECD protein

### Structure of ECD

**Figure 1:**
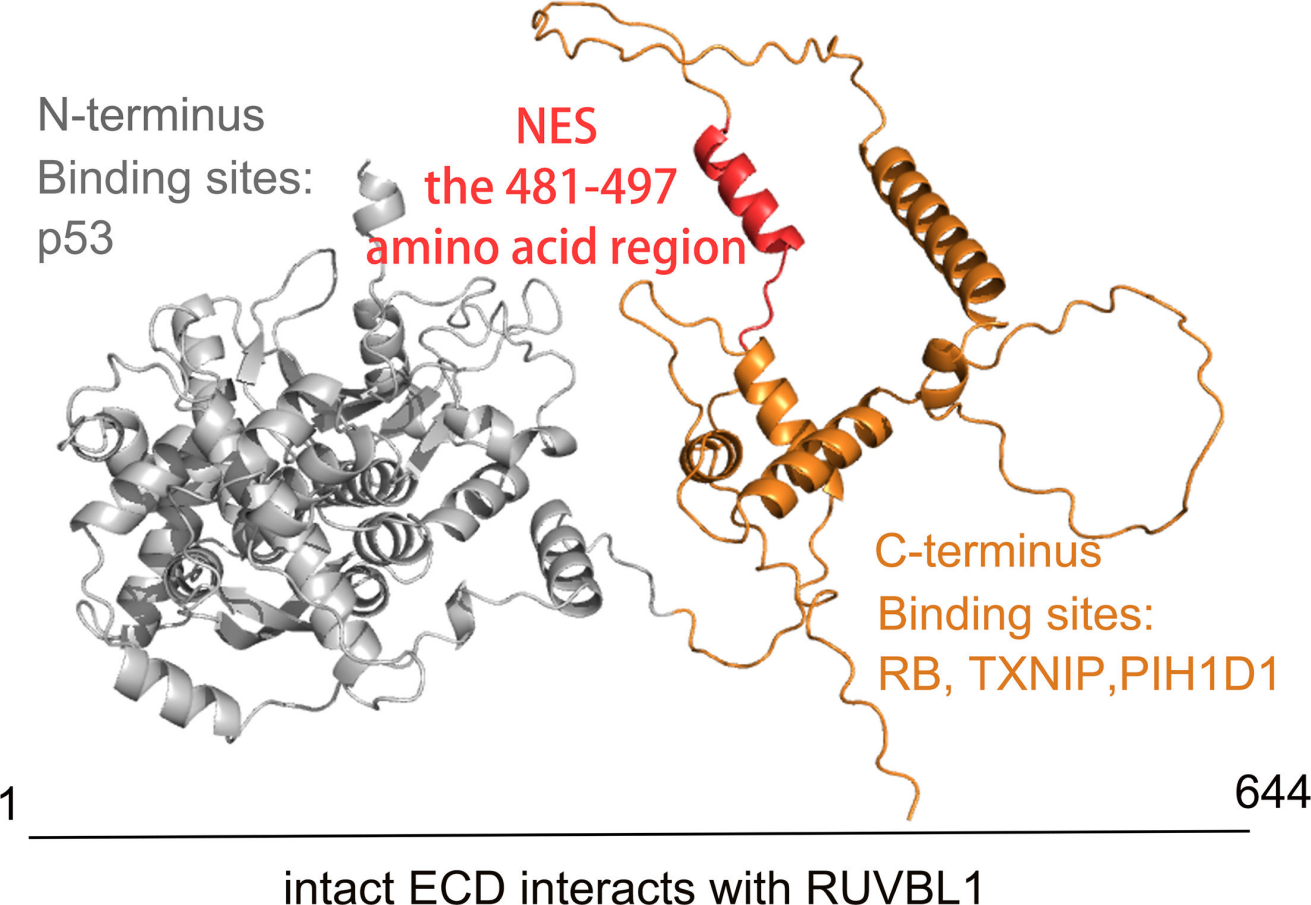
30 Structure of ECD. On the cartoon structure of ECD, the N terminus is in gray, the C-terminus transactivation domain (439–644) in orange, and the potential nuclear export signal (NES, 481–497) in red. The N-terminus interacts with P53, while the C-terminus interacts with RB, TXNIP, and PIH1D1. The intact ECD interacts with RUVBL1. Citations: [8,9,13,14,29,30]. NES, nuclear export signal.

Full-length ECD encoded a protein containing 644 amino acid residues [13,29]. Although the crystal structure of ECD was unknown, circular dichroism measurements and sequence analysis could demonstrate that the full-length ECD mainly has helices [29]. Structural analysis of ECD proteins could be better achieved by searching for proteins that may interact with known motifs. In the absence of binding partners, ECD has a very low melting temperature (Tm) [39.5°C for ECD (1–432) and 41.4°C for ECD (1–534)]; this indicates the presence of loosely folded domains within the ECD fragments. Furthermore, the Kratky plot and *ab initio* bead models revealed the C-terminus of ECD contained sites that bind to several proteins, such as Rb, CK2, and PIH1 domain-containing protein 1 (PIH1D1) [13,29]. These outcomes suggest that ECD serves as a structural hub or scaffolding protein when it combines with other proteins [29]. The full-length structure predicted by AlphaFold V2 [30] was consistent with these results of structural analyses (Figure 1). On the one hand, the p53 binding site was located in the N-terminal region of ECD [9]. On the other hand, only intact ECD could interact with RUVBL1, and deletion in its C- or N-terminal region results in defective binding of ECD to RUVBL1 [13]. RUVBL1 and PIH1D1 belong to the human R2TP complex, and the other two subunits are RPAP3 and RUVBL2 [31,32]. We speculated that ECD may bind to different subunits of the complex to exert its function. Despite the absence of a DNA-binding domain, the C-terminal region of ECD showed a transactivation activity [14]. Strikingly, a single amino acid change at Asp-484 or Leu-489 eliminates the transactivation activity of ECD [14]. However, these outcomes couldn’t provide sufficient information about structural details of ECD.

### Subcellular localization of ECD

ECD is located in both the nucleus and cytoplasm; it does not contain a continuous nuclear localization signal [9,14]. The fragment containing 439–644 amino acids is responsible for its cytoplasmic localization [14]. Kim JH et al. [14] found this fragment strongly supported the transactivation activity of ECD and its transient nuclear localization [14]; the 481–497 amino acid region possibly functioned as a potential nuclear export signal (NES); caused the deletion of the 481–497 amino acid region was initial for cytoplasmic localization of ECD [14]. However, this region does not include a canonical NES. Furthermore, more detailed research could clarify whether the presence of auxiliary proteins is crucial for the process of ECD entering the cell nucleus [13,14,33].

### Function

For comprehensive analysis, we partition ECD’s multifunctionality into six mechanistic domains: (i) cell cycle control, (ii) transcriptional activation, (iii) the production and exportation of RNA, (iv) glucose metabolism, (v) ER stress, and (vi) oncogenic activities. Table 1 systematically integrates published evidence across model systems, detailing phenotypic consequences and underlying molecular pathways to elucidate ECD’s dual roles in normal physiology and disease contexts.

**Table 1:** Models, Phenotypes, and Mechanisms of ECD: A Summary. This table systematically compiles: (i) experimental model systems, (ii) resulting phenotypic manifestations, (iii) elucidated molecular mechanisms, and (iv) source references from the references. Citations: [1–5,7,9–21,23–27]

Model	Phenotype	Mechanism	References
The mutant-ecd^1^-*Drosophila melanogaster*	Larval death; posterior midgut thinning	Eliminates ecdysone biosynthesis enzyme; disrupts the ring gland; impairs U5 snRNP biogenesis	[1–5,7,17,18]
U2OS, MCF-7, H1299 and 21PT-ECD siRNA	Growth inhibition	(with TXNIP) binds Mdm2, inhibiting p53 degradation and stabilizing p53	[9,11]
SGC-7901 and MGC-803-ECD siRNA	Reduced proliferation, migration, invasion	ACK1 overexpression triggers ECD-mediated p53 degradation and activates AKT-POU2F1-ECD signaling	[10,24]
Ecd^fl/fl^ MEFs-adeno-Cre-GFP	Growth inhibition	Interacts with RUVBL1; directly binds Rb’s pocket domain	[12,13]
293T, U2OS-plasmid	Transactivation abrogation	Changes at either Asp-484 or Leu-489	[14]
Prrx1-Cre + Ecd^flox/flox^ mice Prrx1-Cre + Ecd^flox/wt^ mice	Neonatal lethal limb and cranial bone malformations; impaired osteogenesis *in vivo*	Txnip specifically regulates *Tnfsf11* transcription via the Ecd-P300 axis	[15]
MCF10A and 76NTERT -ECD siRNA Ecd^fl/fl^ MEFs-adeno-Cre-GFP	Increase in nuclear poly(A) mRNA signals	Interacts with DDX39A to facilitate mRNA export	[16]
76NTERT-Ecd and/or H-RasQ61L retrovirus	Drives cell cycle, invasion, and tumor growth *in vivo*	Oncogenic cooperation with Ras	[19]
MEF from ECD tg mice	Protects cells from ER stress-induced cell death	Enhances ER stress survival via GRP78 up-regulation and dampening PERK signaling	[20]
CD18/HPAF, Capan1, and MiaPaca-ECD shRNA; injected cells into the pancreas of nude mice	Blocks proliferation, tumorigenesis, and glucose uptake (*in vitro*/*in vivo*)	Regulates expression of GLUT4 and pAkt	[21]
76NTERT-ECD siRNA	Growth inhibition	Leads to widespread RNA splicing aberrations	[23]
SGC-7901 and MGC-803-ECD siRNAs, ECD plasmids; injected cells into NOD-SCID mice	Silencing inhibits, while overexpression promotes, GC cell migration/invasion (*in vitro*) and metastasis (*in vivo*)	Binds hnRNP F, blocking ZFP91-mediated ubiquitination/degradation	[25]
76NTERT and 70NTERT -ECD and/or ERBB2 retrovirus; ECDTg mice	ECD + ERBB2 hMECs showed enhanced migration vs. ECD- or ERBB2-only cells	Disrupts splicing of HPV E6/E7 and oncogenic cellular genes, including core spliceosome components	[26]
ECD tg mice	Exhibits increased susceptibility to heterogeneous tumors	Up-regulates C-MYC and glucose metabolism	[27]

#### Role of ECD in the cell cycle

The tumor suppressor protein p53, which plays a crucial role in cell cycle arrest and apoptosis [34], is critical for reversing the DNA damage-induced G1 phase checkpoint [35]. After DNA damage, p53 is activated and facilitates the elimination or repair of damaged cells, which reduces the risk of mutations [36]. ECD, a novel p53-interacting protein, stabilizes p53 and increases p53-mediated transactivation [9]. Overexpressed ECD promotes p53 accumulation and facilitates p53-dependent acceleration of cellular senescence; however, ECD does not enhance p53 transcription by interacting with p53-targeted promoter [14]. The Mdm2 is an E3 ligase that targets p53 for its proteasomal degradation. Alternatively, ECD enhances p53 protein levels by suppressing the Mdm2-mediated degradation of p53 [37]. Moreover, Inpyo Choi et al. found that the C-terminal region of ECD interacts with TXNIP, a well-known tumor-suppressing factor [11]. They found the ectopic overexpression of TXNIP and ECD, or the co-overexpression of TXNIP and ECD, inhibited the interaction of Mdm2 with p53 and increased the stability of p53 [11]. TXNIP–ECD interaction decreases Mdm2-mediated p53 ubiquitination and enhances the stability and activity of p53. ECD possibly masks ubiquitination sites on p53 or facilitates the recruitment of deubiquitinating enzymes [9]. Meanwhile, elevated expression of activated Cdc42-associated kinase 1 (ACK1) promotes ECD overexpression and induces p53 ubiquitination [24]. ACK1 acts as an activated transmembrane effector of receptor tyrosine kinases (RTKs) and transmits various RTK signals, which is a carcinogenic factor [38]. We speculated that ECD plays various roles in regulating p53 expression in different cell types; however, the underlying regulatory mechanism needs to be further investigated.

**Figure 2:**
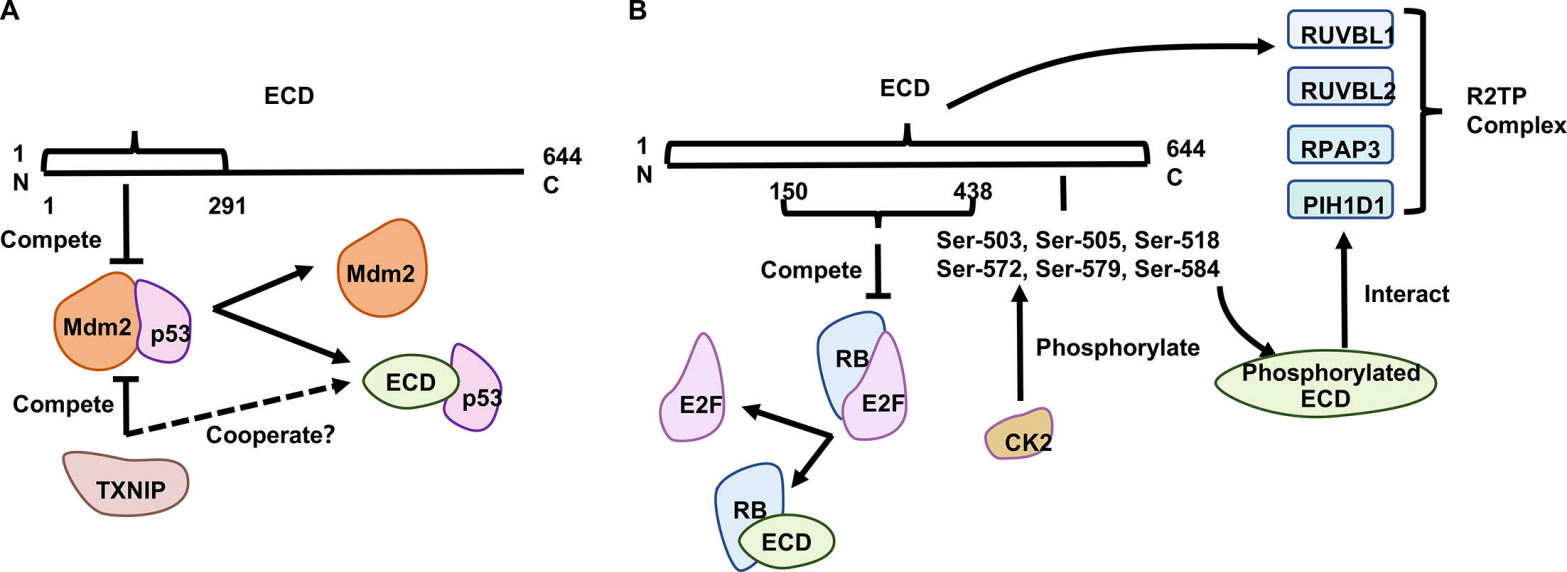
ECD in Cell Cycle Regulation. (**A**) ECD interacts with p53 by inhibiting the Mdm2-mediated degradation of p53. ECD (amino acids 1–291) can interact with p53. Besides, the interaction between ECD and Txnip or antagonism of Mdm2 by Txnip alone can decrease the ubiquitination of p53 mediated by Mdm2. (**B**) ECD (amino acids 150–438) competes with E2F to bind hypophosphorylated Rb at its pocket domain. CK2 phosphorylates ECD at two clusters (including proximal clusters of Ser-503, Ser-505, and Ser-518 and distal clusters of Ser-572, Ser-579, and Ser-584). Phosphorylated ECD is recognized by the PIH-N structural domain of PIH1D1 to be recruited into the R2TP complex. The mutations of Ser-505 and Ser-518 in ECD can eliminate the interaction of ECD with PIH1D1. Full-length ECD interacts with RUVBL1 to regulate the cell cycle progression. Citations: [9,11–13].

The RB protein family includes essential regulators of cell growth, and the transcription factor E2F is crucial for G1/S transition [39,40]. ECD (150–438 amino acid region) competes with E2F that binds to the hypophosphorylated RB in its pocket domain [12]. Then free E2F will up-regulate the expression of target genes to promote cell cycle progression [41–43]. The team also found ECD interacted with RUVBL1 and promoted cell cycle progression; this interaction is independent of the phosphorylation of ECD or its association with PIH1D1 [13]. Both proteins belong to the R2TP complex, an HSP90 cochaperone, which participates in many biological processes, including chromatin remodeling, transcriptional regulation, ribonucleoprotein complex biogenesis, and the DNA damage response [44,45]. Interestingly, the deletion in either ECD or RUVBL1 is associated with early embryonic lethality, and the knockdown (KD) of either ECD or RUVBL1 results in G1/S cell cycle arrest [12,46,47]. Both proteins exhibit many similarities in their cellular phenotypes. In addition, cell cycle progression needs the interaction of ECD and RUVBL1, and its interaction with RB alone cannot sufficiently facilitate this function [13]. Furthermore, the conserved DpSDD motif in ECD can be phosphorylated by CK-2, recognized by the PIH-N structural domain of PIH1D1, and thus, ECD is recruited into the R2TP complex [48] . There are two potential serine clusters in the C-terminal region of ECD (proximal clusters of Ser-503, Ser-505, and Ser-518 and distal clusters of Ser-572, Ser-579, and Ser-584), both of which can be phosphorylated by CK2; phosphorylation is more frequent specifically in the proximal cluster than in the other [13]. Mutations in Ser-505 and Ser-518 in ECD suppress the interaction between ECD and PIH1D1 [13,48]. Although the specific mechanism by which ECD binds to PIH1D1 is unclear, the rescue function of the 6 S/A mutant (S503, S505, S518, S572, S579, and S584; designated 6 S/A) is significantly weaker than that of the 3 S/A mutant (S503A, S505A, and S518A), which further highlights the importance of ECD phosphorylation [13]. Thus, ECD appears to act synergistically with multiple proteins to regulate cell cycle progression (Figure 2).

#### Role of ECD in transactivation

**Figure 3:**
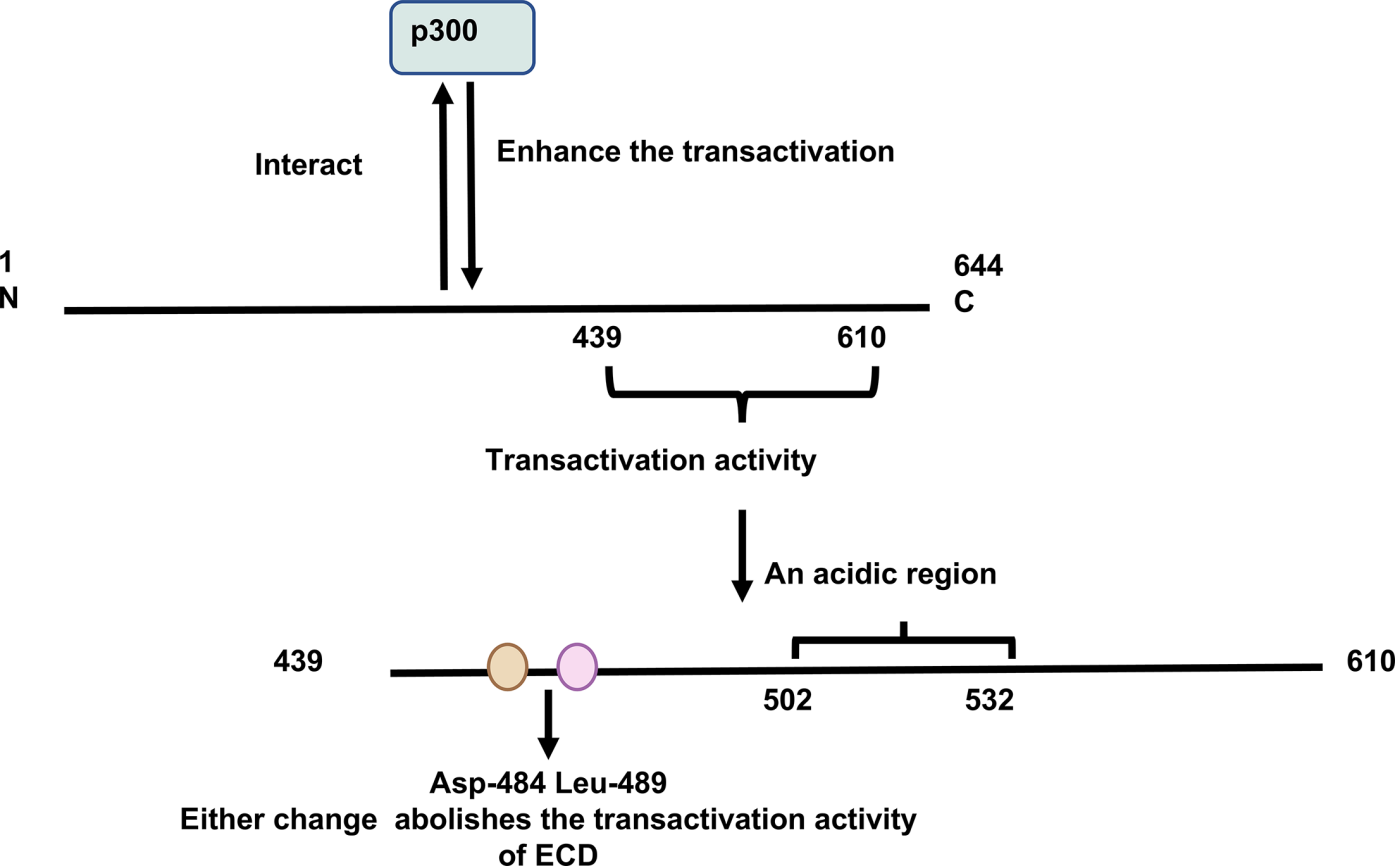
ECD in Transactivation. ECD interacts with p300 to enhance the transactivation mediated by ECD. Txnip specifically modulates *Tnfsf11* transcription through the Ecd-P300 axis. The region (amino acids 439–610) has a transactivation activity in its C-terminal. A single amino acid change at either Asp-484 or Leu-489 essentially completely abolishes the transactivation activity of ECD. The fragment of amino acids 502–532 of ECD, an acidic region, is usually related to transactivation functions. Citations: [14,15].

Preliminary evidence suggested ECD could act as a transcriptional activator, potentially stimulating glycolytic pathway activity through enhanced expression of metabolic enzymes [8]. ECD, lacking a DNA-binding domain, does not potentially serve as a primary transcription factor; however, its C-terminal region (amino acids 439–610) has high transactivation activity (Figure 3) [14]. The nuclear positioning of ECD is brief; however, it is sufficient for the transactivation activity. Notably, the N-terminal region (amino acids 1–438) of ECD may suppress its voluntary transactivation activity of the C-terminus through altered folding of the protein, as reported in the case of FoxM1, a transcription factor [14,49]. Asp-484 and Leu-489 appear to be the most crucial residues for this transactivation activity; any change in either of these two amino acids inhibits the transactivation activity (Figure 3) [14]. The fragment containing amino acids 502–532 represents an acidic region of ECD, which is possibly vital for its potential transactivation activity [14]. Previous articles have reported that acidic regions are usually related to transactivation functions [50]. Moreover, p300 is a transcription co-activator that binds to certain transcription factors and interacts with other co-activators to up-regulate the expression of target genes [51]; it interacts with ECD and enhances the transactivation activity (Figure 3) [14]. In our research, we found Txnip specifically governed *Tnfsf11* transcriptional regulation through the Ecd-P300 signaling axis [15]. Complete genetic ablation of *Ecd* in bone marrow stromal cells leads to defective osteogenesis *in vivo*, manifesting as severe developmental abnormalities in limb and cranial bone formation that culminate in neonatal lethality.

#### Role of ECD in regulating the production and exportation of RNA

**Figure 4:**
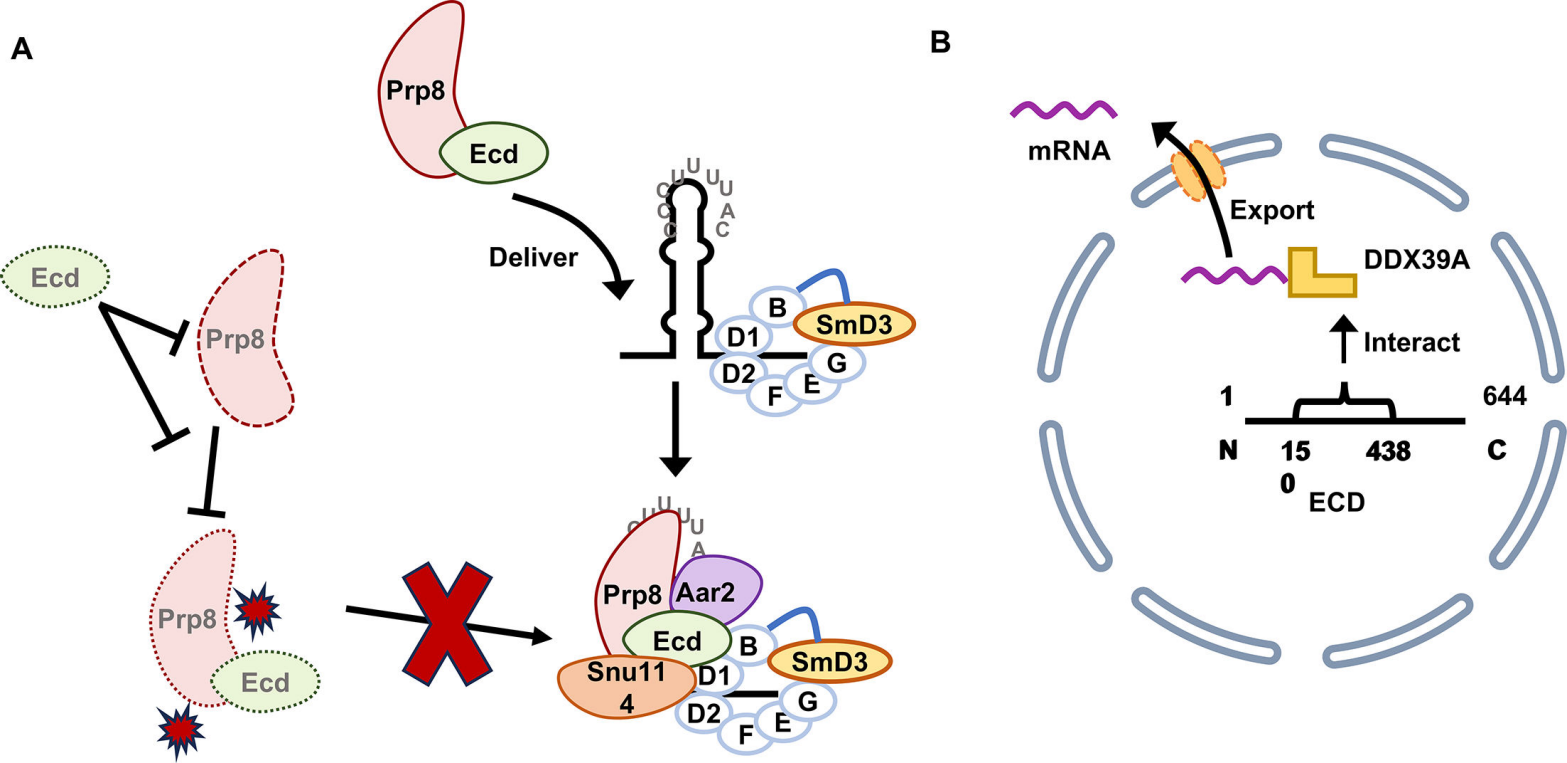
ECD in RNA Production and Export. (**A**) ECD protects PRP8 from degradation and facilitates the transport of PRP8 to SmD3, thereby promoting the biogenesis of U5 snRNP. Notably, the absence of ECD also impairs the binding of PRP8 to SmD3. (**B**) ECD (amino acids 150–438) interacts with DDX39A to modulate mRNA shuttle. Citations: [16–18,23,26].

During the splicing of the pre-mRNA, the excision of introns from the nascent pre-mRNA and accurate fusion of exons process are very crucial for gene function [52]. The spliceosome is mainly composed of five main building blocks: U1, U2, U4, U5, and U6 [53]. U snRNPs and other proteins, such as the DEAD and DEAH-box proteins, are dynamically related to this complex [54]. Each snRNP consists of a short noncoding uridine-rich RNA (*U snRNA*), a common heptameric Sm or Sm-like protein ring, and a set of specific proteins [55,56]. The splicing factor Prp8, one of the largest and most conserved proteins across the classes of spliceosomes [57], is involved in the core U5 snRNP and forms the precursor U5 snRNP with Aar2 and Snu114 [58,59]. Therefore, when proteins involved in the formation of U snRNPs undergo variants, they are associated with various diseases, as the spliceosome is essential in all cells and at all stages of development [60]. Taking U5 snRNP as an example, variants of TXNL4A and EFTUD2 are associated with craniofacial malformations, while variants of PRPF8 and SNRNP200 are associated with retinitis pigmentosa. Additionally, alterations in the protein levels of PRPF8, EFTUD2, and SNRNP200 affect their ability to regulate alternative splicing (AS), representing a novel mechanism by which viruses modulate cellular AS [60,61]. In *Drosophila*, Ecd directly interacts with the core components of the U5 snRNP spliceosome complex, including Prp8 [17,18]. Then, Ecd delivers Prp8 to U5 snRNP via the formation of a complex with SmD3 (Figure 4). Loss of Ecd reduces Prp8 protein levels and compromises U5 snRNP biogenesis, which affects the splicing of a large intron in the *CYP307A2/spookier (spok*) pre-mRNA, leading to the inhibited production of this important ecdysteroid biosynthetic enzyme essential for *Drosophila* development [17,18]. Notably, ECD associates with subunits of the R2TP/Prefoldin-like co-chaperone complex involved in the assembly of functional U5 snRNPs [13,62], which implies that ECD is a possible molecular linker that connects the R2TP complex and U5 snRNP biogenesis. In the 76N.TERT cell line, depletion of *ECD* induces global disturbances in RNA splicing fidelity and significantly alters the binding dynamics of U5 snRNA, which demonstrates ECD plays a role in RNA splicing in mammals [23,26]. The Vimla Band group recently demonstrated in human cell lines that ECD interacts directly with RNA through its amino acid residues 135–148, as well as specifically binds U5 snRNA, according to their preprint [19].

The researchers also found a variety of proteins involved in mRNA export in the interacting proteins of ECD when they analyzed them [9,12,13,27]. ATP-dependent RNA helicase DDX39A is crucial for mRNA export [18]; the interaction of ECD (amino acids 150–438) and DDX39A is associated with this mechanism of mRNA export (Figure 4) [16]. The depletion of *ECD* does not alter the expression level or subcellular localization of DDX39A or its associated proteins, and while DDX39A cannot functionally compensate for the mRNA export deficiency caused by ECD loss, these two factors co-operate to regulate mRNA export, with ECD maintaining functions that are independent of DDX39A [16]. As mentioned in previous reviews, pre-mRNA splicing is not an isolated event but is closely related to transcription elongation, quality control, nuclear export, and RNA product degradation [63]. In *Drosophila*, Ecd has been found to interact with the core components of U5 snRNP and to influence its expression level, thereby affecting the stability of the spliceosome and causing problems with RNA splicing[17,18]. In mammals, ECD has been shown to possess transactivating activity and interacts with nuclear export proteins[14,16]. Additionally, ECD localizes between the cytoplasm and nucleus[9,14], and its presence has been detected in a complex containing human PRPF8[64]. We anticipate that future research will further elucidate the role and mechanistic basis of ECD in RNA splicing, paving the way for its potential applications in the diagnosis and treatment of related diseases.

#### Role of ECD in glucose metabolism

ECD can rescue the growth defect and glycolytic phenotype caused by the Gcr2 mutation in *Saccharomyces cerevisiae*; hence, it is considered a homologous analog of Gcr2 in humans [8]. However, the lack of structural homology of ECD proteins to Gcr2 and the absence of a true ECD homologue in *S. cerevisiae* suggest that ECD may function through a different mechanism [12]. In recent decades, the role of ECD in glucose metabolism has been studied. In transgenic mice with mammary epithelium-targeted overexpression of an inducible ECD transgene (ECDTg), mammary hyperplasia, preneoplastic lesions, and heterogeneous tumors with occasional lung metastases were reported [27]. RNA-Seq analysis of ECDTg tumors revealed a c-MYC signature and altered ECD levels, regulating c-MYC mRNA and protein levels, as well as glucose metabolism [27]. The overexpression of ECD and c-MYC in mice rescued the ECD KD-associated suppressed glucose uptake. These results indicate that ECD has a conserved function in controlling glucose metabolism in various taxa ranging from yeast to mammals, possibly through its transactivation function regulating genes significantly involved in the metabolic network [21,27].

#### Role of ECD in ER stress

The ER is a major organelle involved in protein synthesis [65]. In the organism, conserved ER transmembrane sensors, such as inositol-acquiring enzyme 1α, PERK, and activating transcription factor 6, monitor abnormal ER function to maintain homeostasis of protein synthesis. These sensor-activated steady-state signaling pathways are known as the unfolded protein response (UPR) [66,67]. During ER stress, the load of unfolded proteins increases, and competition for binding to GRP78 separates it from PERK, leading to the activation of the PERK pathway that can attenuate the negative outcomes of ER stress [68]. ECD increases GRP78 protein levels during ER stress and prevents the separation of GRP78 from PERK, thereby promoting cell survival via impaired activation of the PERK signaling pathway. Additionally, ER stress decreased the level of ECD protein [20].

#### Role of ECD in cancers

Cancer comprises a collection of diseases driven by dysregulated cell proliferation. Under physiological conditions, cell division is tightly controlled by evolutionarily conserved cell cycle regulatory mechanisms to ensure the faithful generation of genetically identical daughter cells. Disruption of these control systems results in uncontrolled proliferation, genomic instability, and malignant transformation, ultimately culminating in carcinogenesis [69]. While previous studies have established the critical role of ECD in cell cycle regulation, its potential involvement in oncogenesis remains to be fully elucidated [12,13]. To investigate this potential association, researchers systematically analyzed ECD expression patterns across multiple cancer types. They found ECD was highly expressed in many cancers and associated with poor prognostic outcomes [10,21–25].

**Figure 5:**
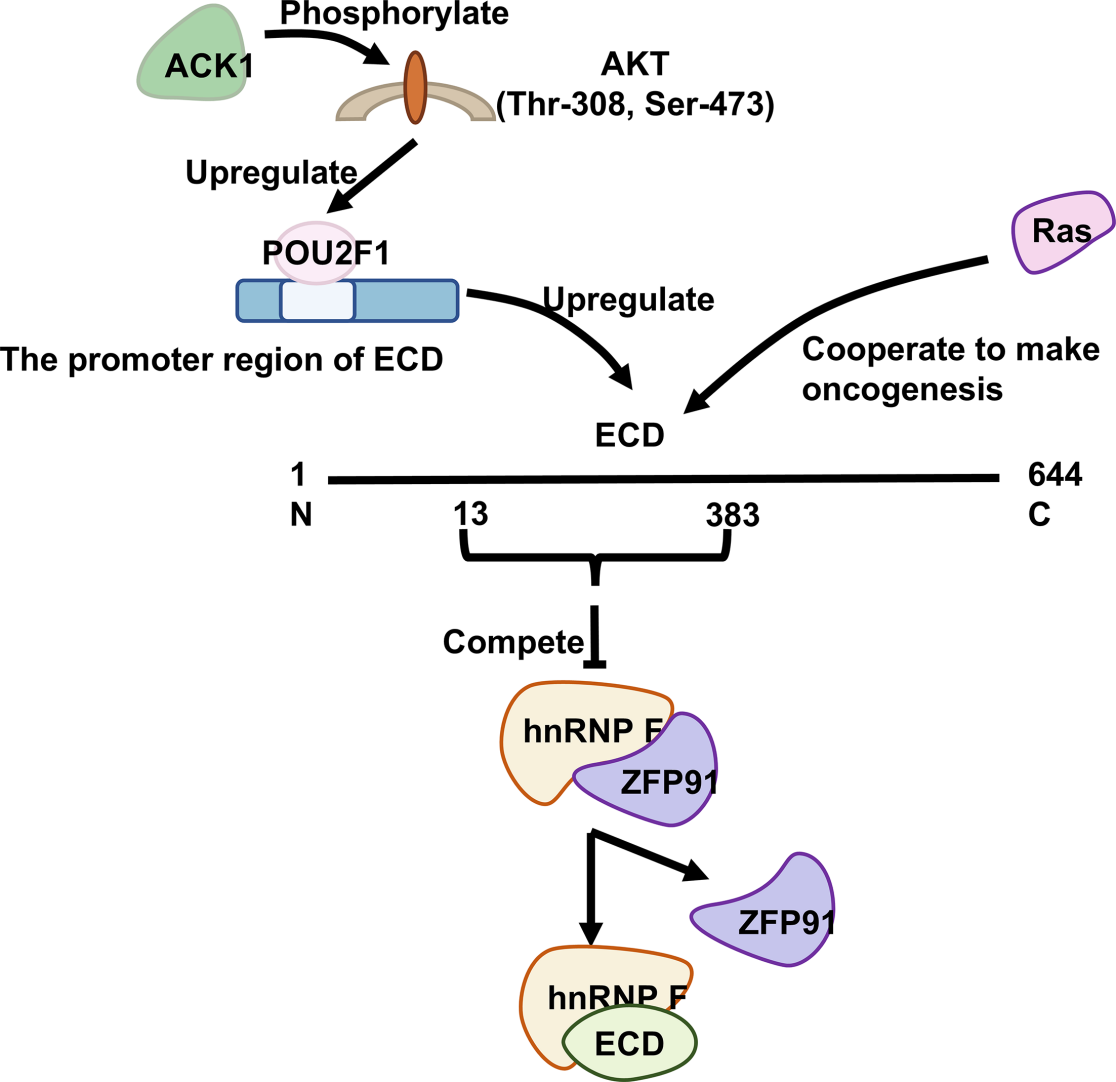
ECD in Cancers. ACK1 phosphorylates AKT at Thr-308 and Ser-473. The AKT pathway is activated to up-regulate POU2F1, which regulates ECD expression in gastric cancer cells by directly binding to the promoter region of ECD. ECD interacts with mutant RAS to promote cancer development. ECD (amino acids 13–383) competitively binds to hnRNP F, thereby preventing hnRNP F from interacting with ZFP91 and preventing ZFP91-mediated ubiquitination and proteasomal degradation of hnRNP F. Citations: [10,23–25,38]. hnRNP F, heterogeneous nuclear ribonucleoprotein F.

In breast cancer, Vimla Band et al. found the expression of ECD positively correlated with HER2/neu overexpression, a known sign of poor prognosis [22]. However, ECD expression is unrelated to ER/PR status and hormone receptors that predict the response to endocrine therapy [22]. In ErbB2 (a driver oncogene) positive breast cancer cells, ECD controls ErbB2 mRNA export and stability by regulating the decreased expression of herstatin (a tumor suppressor splice variant) and increased expression of the Δ16HER2 variant (a pro-oncogenic splice variant) [16,70–72]. To further understand the role of ECD in the development of breast cancer, Vimla Band et al. found knocked down ECD in human mammary epithelial cells and observed cycle arrest [23]. At the same time, the researchers established stable cell lines with ECD overexpression. Given the established crosstalk between Rat Sarcoma Viral Oncogene Homolog (RAS) activation and ErbB2 (HER2) signaling in cell cycle regulation and considering the high frequency of H-RAS mutations in cooperative oncogenesis, they further generated dual-overexpression systems co-expressing ECD and constitutively active RAS [22,23,73]. Overexpression of ECD and RAS results in improved survival in the absence of epidermal growth factor and the ability of invasion and migration through the significant up-regulation of the autophagy marker protein LC3-II. And the process of autophagy promotes cell survival during nutrient starvation, hypoxia, and other stresses [74]. Specifically, ECD cooperates with RAS to stimulate the downstream effector Phosphorylated Extracellular signal-Regulated Kinase (p-ERK), a physiological regulator of RAS-mediated cell proliferation (Figure 5) [23]. The KD of either ECD or DDX39A decreases the export of ErbB2 mRNA from the nucleus to the cytoplasm. Significantly reduced cell proliferation, colony formation, anchorage-dependent growth, migration, and invasive ability of cancer cells are evident in the absence of ECD [16]. Their recent preprint demonstrates that ECD directly interacts with the mRNA of key glycolytic enzymes (LDHA, PKM2, HK2) and the UPR regulator GRP78. By stabilizing these mRNAs, ECD enhances glycolysis and the UPR, thereby driving tumor cell proliferation [28]. In addition, the expression of ECD significantly increased with an increase in dysplasia. In the ECD-positive primary tumor, the metastatic site was more likely to be ECD-positive. However, notably, the expression of ECD was negatively correlated with the degree of differentiation, and no significant difference in intensity was identified between different stages of differentiation [21]. Moreover, ECD-negative primary tumors are characterized by metastatic lesions that do not express ECD. ECD KD inhibits cell growth *in vitro* and tumorigenicity of pancreatic cancer cells in orthotopic tumors in mice. However, no significant change in cell death indicates that differences in cell proliferation are possibly the primary reason for cell growth [21].

In pancreatic cancer tissues, ECD was also overexpressed [21]. The KD of ECD induced decreased glucose uptake in cells, a disordered cell state at basal levels, and insulin stimulation [21]. After insulin stimulation, the transport of glucose transporter 4 (GLUT4)-containing vesicles to the membrane is delayed in ECD-KD cells [21]. GLUT4 belongs to the glucose transporter family and is responsible for glucose transport between the extracellular space and the cytoplasm, which is realized by GLUT4-storage vesicles (GSVs) [75]. It was further shown that the GLUT4 transcriptional regulator, GLUT4 enhancer factor, and protein expression are down-regulated after the KD of ECD in pancreatic cancer cells [21]. Moreover, phosphorylation of protein kinase B (Akt), a serine/threonine kinase and a major multifunctional node in the insulin signaling pathway, was decreased in tumor lysates of ECD-KD mice. Previous studies have shown that Akt, a key regulator of GLUT4 expression and the glycolytic pathway in cancer cells [76,77], can phosphorylate AS160, a Rab-GTPase activating protein (GAP), to eliminate the activity of GAP, enabling GTP to load onto Rab molecules and facilitating the transport of GSVs from inside the cell to the cell membrane [78,79]. Moreover, Akt phosphorylates TXNIP at residue S308, which is necessary for Akt-induced glucose uptake by primary MEFs [80]. On the other hand, researchers found that once disrupted TXNIP–GLUT4 interactions by inducing TXNIP phosphorylation during insulin stimulation will reduce GLUT4 endocytosis [80,81]. The aforementioned interacting proteins are all involved in sugar metabolism and the cell cycle. Therefore, researchers speculate that ECD is a key molecule between the cell cycle and metabolic signaling, as it was identified in yeast to rescue sugar glycolysis-deficient mutants [8,21].

In gastric cancer (GC), ECD overexpression is associated with poor prognosis. ECD promotes GC invasion and translocation by blocking heterogeneous nuclear ribonucleoprotein F (hnRNP F) ubiquitination and degradation (Figure 5) [25]. hnRNP F, which belongs to the hnRNP and is altered in many cancers, is an RNA-binding protein that regulates multiple aspects of nucleic acid metabolism, including transcription, translation, splicing, and mRNA stabilization [82,83]. ECD competes with ZFP91, an E3 ubiquitin ligase responsible for hnRNP F ubiquitination at Lys-185 and proteasomal degradation, for binding to hnRNP F through the N-terminal STG1 domain (13–383 amino acids) [84,85]. At the transcriptional level, ECD plays an essential downstream role in the ACK1-AKT-POU class 2 homeobox 1 (POU2F1) axis, leading to promoted GC metastasis and epithelial-mesenchymal transition (Figure 5) [24]. First, AKT, a serine/threonine protein kinase localized in the plasma membrane, is phosphorylated by ACK1 at Thr-308 and Ser-473. The activated AKT pathway up-regulates the transcription factor POU2F1 that binds to a cis-acting octamer element to regulate the transcription of target genes [86]. POU2F1 regulates ECD expression in GC cells by directly binding to the promoter region of ECD. This pathway is evident in clinical GC specimens; the phosphorylation levels of AKT at Thr-308 and Ser-473 and the levels of POU2F1 and ECD are positively associated with ACK1 levels [24].

Overexpression of ECD in cervical, head, and neck squamous cell carcinoma (HNSCC) cell lines and tumor tissues indicates shorter survival of patients [26]. ECD is essentially associated with the maintenance of the oncogenic properties, such as proliferation rate, anchorage-independent growth, cell migration, and invasion, in cervical cancer cells [26]. High-risk human papillomaviruses (HPVs) are causally associated with anogenital, skin, and upper respiratory tract cancers. ECD selectively binds to high-risk (HPV 16 or 18) rather than low-risk HPV E6 proteins (HPV 6 or 11) through its C-terminus (amino acids 493–644); E6 does not target ECD for degradation, although E6 mediates the degradation of multiple target proteins, including p53, via its interaction with E6AP [9,26,87]. In addition, ECD can cooperate with HPV E7 to facilitate the immortalization of keratinocytes. Notably, ECD KD showed a decreased level of the E7 tumor protein, whereas the level of RB increased, suggesting that ECD-mediated regulation of E7 expression is independent of E6/E7 splicing [26]. ECD KD revealed altered mRNA levels of genes involved in several oncogenic pathways, including EIF2, mTOR, PI3K/AKT signaling pathways, and classical splicing factors, such as SRSF3 (SRp20) [26]. One of the key pathways activated during HPV+HNSCC and cervical cancer oncogenesis is the mTOR pathway [88]. The gene-expressing SRSF3, which is highly expressed in cervical cancer and is essential for the maintenance of cell growth and transformation, regulates HPV RNA splicing [89]. Targeting ECD can play an important role in various disease processes, including cell growth and survival, glucose metabolism, and tumorigenesis. Therefore, further preclinical and clinical studies are needed to decipher the detailed molecular mechanism underlying the correlation of ECD with the pathogenesis and progression of specific diseases, which can help identify novel therapeutic targets.

Consistent with previous findings, ECD demonstrates ubiquitous overexpression across multiple tumor types, where it functionally intersects with cell cycle regulation, RNA splicing machinery, and UPR homeostasis [10,16,21–24]. For example, during the growth of tumor tissues, their oncogenic activation alters chromosome counts, and increased secretory ability increases the demand for protein production [90]. Moreover, external factors, such as hypoxia and acidosis, interfere with ER homeostasis. All these tumor-related factors contribute to ER stress, while these conditions will influence ECD [91]. This is likely one of the reasons for the high expression of ECD in cancers.

## Conclusion

ECD is a highly conserved protein that plays crucial roles in various physiological and pathophysiological processes. Sadly, despite the importance of its function, there are not many relevant articles, and the exploration of the mechanism has not been comprehensive in these past decades. Therefore, we hope that this review will provide a more systematic introduction to ECD and provide a reference for the exploration of its functions and mechanisms. Based on the binding site, ECD-interacting proteins can be broadly classified into two categories: proteins that bind to the C-terminus and those that bind to the N-terminus. Most proteins bind to the C-terminus of ECD; this is apparently attributable to the disordered structure of the C-terminus, which can adopt various conformations suitable for binding other proteins [14]. We know that ECD is involved in physiological processes, such as cell cycle regulation, transcriptional activation, modulation of RNA production and export, ER stress, and glucose metabolism [12,13,16,18,27]. Furthermore, it is highly expressed in some pathological processes, which correlate with poor clinical outcomes, such as cancer [10,23–25,38]. These physiological and pathological processes influence each other. For example, impaired mRNA nuclear export, disturbed ER stress, and dysregulated glucose metabolism are associated with the development of cancer [16,74,92–94]. Critically, ECD has been identified as a bifunctional regulator participating in both RNA splicing modulation and transcriptional activation [19]. Emerging evidence from a preprint study reveals that ECD directly binds RNA, with particular specificity for U5 snRNA. Such finely tuned molecular control plays a pivotal role in preserving proper cellular function. Therefore, comprehensive knowledge about the association of ECD with pre-mRNA splicing in mammals, as well as the mechanisms and interacting proteins involved with ECD-regulated cell cycle, transactivation, and mRNA export is crucial for understanding the physiological and pathological involvement of ECD, which can prospectively suggest new directions for clinical therapy. Considering the important role of ECD in cell growth, a targeted inhibitor that can find its specific site would be a better solution to the infinite proliferation of tumor cells.

## Data Availability

Data and materials are provided in the references.

## Abbreviations

ACK1: activated Cdc42-associated kinase 1
AS: alternative splicing
Akt: protein kinase B
CK2: Casein Kinase 2
DDX39A: DExD-box helicase 39A/ DEAD-box helicase 39A
ECD: Ecdysoneless
ECDTg: ecdysoneless transgene
EFTUD2: elongation factor Tu GTP binding domain containing 2
EIF2: eukaryotic translation initiation factor 2
ER: Estrogen Receptor
ER: endoplasmic reticulum
ErbB2: Erythroblastic Leukemia Viral Oncogene Homolog 2
GAP: GTPase activating protein
GC: gastric cancer
GLUT4: glucose transporter 4
GRP78: glucose-regulated protein 78
GSV: glucose transporter 4-storage vesicle
HER2/neu: Human Epidermal Growth Factor Receptor 2
HK2: hexokinase 2
HNSCC: head and neck squamous cell carcinoma
HPVs: human papillomaviruses
KD: knockdown
LDHA: lactate dehydrogenase A
Mdm2: murine double minute-2
NES: nuclear export signal
P53: tumor protein p53
PIH1D1: PIH1 domain-containing protein 1
PI3K: phosphatidylinositol 3-kinase
PKM2: pyruvate kinase M2
POU2F1: POU class 2 homeobox 1
PR: Progesterone Receptor
PRP8: Pre-mRNA Processing Factor 8
RAS: Rat Sarcoma Viral Oncogene Homolog
RB: Retinoblastoma protein
RPAP3: RNA polymerase II-associated protein 3
RTKs: receptor tyrosine kinases
RUVBL: RuvB like AAA ATPase
RUVBL1: RuvB like AAA ATPase 1
*SGT1*: human suppressor of GCR two
SRSF3: serine and arginine rich splicing factor 3
TERT: telomerase reverse transcriptase
TXNIP: thioredoxin-interacting protein
TXNL4A: thioredoxin like 4A
Tnfsf11: Tumor Necrosis Factor Superfamily Member 11
UPR: unfolded protein response
U snRNA: uridine-rich RNA
ZFP91: zinc finger protein 91
hnRNP F: heterogeneous nuclear ribonucleoprotein F
p-ERK: Phosphorylated Extracellular signal-Regulated Kinase
snRNPs: small nuclear ribonucleoprotein particles
